# A simple scoring model based on machine learning predicts intravenous immunoglobulin resistance in Kawasaki disease

**DOI:** 10.1007/s10067-023-06502-1

**Published:** 2023-01-11

**Authors:** Yuto Sunaga, Atsushi Watanabe, Nobuyuki Katsumata, Takako Toda, Masashi Yoshizawa, Yosuke Kono, Yohei Hasebe, Keiichi Koizumi, Minako Hoshiai, Eiryo Kawakami, Takeshi Inukai

**Affiliations:** 1 Faculty of Medicine, Department of Pediatrics, University of Yamanashi, 1110 Shimokato, Chuo, Yamanashi 409-3898 Japan; 2 Department of Neonatology, Yamanashi Prefectural Central Hospital, Kofu, Yamanashi Japan; 3 Department of Pediatric Cardiology, National Cerebral and Cardiovascular Center, Osaka, Japan; 4 Department of Pediatrics, Fujiyoshida Municipal Hospital, Fujiyoshida, Yamanashi Japan; 5 Department of Pediatrics, Yamanashi Prefectural Central Hospital, Kofu, Yamanashi Japan; 6 Artificial Intelligence Medicine, Graduate School of Medicine, Chiba University, Chiba, Japan; 7 Institute for Advanced Academic Research (IAAR), Chiba University, Chiba, Japan; 8 Advanced Data Science Project, RIKEN Information R&D and Strategy Headquarters, Kanagawa, Japan

**Keywords:** Intravenous immunoglobulin resistance, Kawasaki disease, Machine learning, Shapley additive explanation

## Abstract

**Introduction:**

In Kawasaki disease (KD), accurate prediction of intravenous immunoglobulin (IVIG) resistance is crucial to reduce a risk for developing coronary artery lesions.

**Objective:**

To establish a simple scoring model predicting IVIG resistance in KD patients based on the machine learning model.

**Methods:**

A retrospective cohort study of 1002 KD patients diagnosed at 12 facilities for 10 years, in which 22.7% were resistant to initial IVIG treatment. We performed machine learning with diverse models using 30 clinical variables at diagnosis in 801 and 201 cases for training and test datasets, respectively. SHAP was applied to identify the variables that influenced the prediction model. A scoring model was designed using the influential clinical variables based on the Shapley additive explanation results.

**Results:**

Light gradient boosting machine model accurately predicted IVIG resistance (area under the receiver operating characteristic curve (AUC), 0.78; sensitivity, 0.50; specificity, 0.88). Next, using top three influential features (days of illness at initial therapy, serum levels of C-reactive protein, and total cholesterol), we designed a simple scoring system. In spite of its simplicity, it predicted IVIG resistance (AUC, 0.72; sensitivity, 0.49; specificity, 0.82) as accurately as machine learning models. Moreover, accuracy of our scoring system with three clinical features was almost identical to that of Gunma score with seven clinical features (AUC, 0.73; sensitivity, 0.53; specificity, 0.83), a well-known logistic regression scoring model.

**Conclusion:**

A simple scoring system based on the findings in machine learning seems to be a useful tool to accurately predict IVIG resistance in KD patients.

**Supplementary Information:**

The online version contains supplementary material available at 10.1007/s10067-023-06502-1.

**Table:** 

**Key Points** *• In Kawasaki disease (KD), accurate prediction of intravenous immunoglobulin (IVIG) resistance is crucial to reduce a risk for developing coronary artery lesions.* *• Machine learning model predicted IVIG resistance in KD patients, and Shapley additive explanation (SHAP) was a useful approach for explaining the outcome of the machine learning model.* *• A simple scoring system using three clinical features (days of illness at initial therapy, serum levels of CRP, and total cholesterol at diagnosis) based on SHAP efficiently predicted IVIG resistance.*	

## Introduction

Kawasaki disease (KD) is an acute febrile illness in infants and children. Of clinical importance, it is characterized by systemic vasculitis and affects medium-sized arteries, especially the coronary arteries [1, 2]. To avoid the development of coronary artery lesions (CAL), high-dose (2 g/kg) intravenous immunoglobulin (IVIG) therapy has been established as a standard initial treatment for KD patients in the acute phase [2, 3]. However, approximately 20% of KD patients are resistant to the initial IVIG treatment [3], and IVIG resistance is a typical risk factor for developing CAL [1, 4–7]. Under these circumstances, several recent studies showed a possible clinical benefit of intensive initial IVIG therapy combined with other anti-inflammatory agents for the high-risk KD patients [6, 8–10]. For effective pre-treatment risk stratification, it is crucial to establish a scoring system to accurately predict IVIG resistance at the timing of clinical diagnosis of KD. Currently, there are several widely used Japanese scoring models for predicting IVIG resistance: Gunma score proposed by Kobayashi et al. [11], Kurume score proposed by Egami et al. [12], and Osaka score proposed by Sano et al. [13]. These scoring systems were developed by the logistic regression analysis of clinical profiles and laboratory findings before initial treatment, which were selected based on statistical assumptions.

To establish a more reliable and simple scoring system for the prediction of IVIG resistance in KD patients, an alternative approach using large data repositories is required. Recently developed machine learning approach has shown great potential for assisting the clinical diagnosis and predicting outcomes [14–19]. Machine learning utilizes sophisticated algorithms operating on large-scale, heterogeneous datasets to uncover informative patterns that would be difficult or impossible for even well-trained individuals to identify [20]. Indeed, several recent studies applied machine learning to predict IVIG resistance in KD patients and confirmed its usefulness [14–16]. However, in clinical practice, even if machine learning has a high degree of accuracy, a simple scoring system is more convenient for risk-stratified treatment.

In the present study, we applied machine learning to predict IVIG resistance in 1002 KD cases treated with single IVIG protocol in multiple institutes. Subsequently, using the three most important features associated with IVIG resistance in the machine learning, we developed a new simple scoring system and confirmed its utility by comparison with the three representative scoring systems.

## Materials and methods

### Study participants

The study is a retrospective review of multicenter registration database of 1141 consecutively diagnosed KD patients who were diagnosed between June 2010 and December 2020 in 12 inpatient facilities for the care of pediatric patients, as listed in Supplemental Table 1. Diagnosis of KD was retrospectively confirmed based on criteria defined in the fifth edition of the Japanese Kawasaki Disease Diagnostic Guidelines [21]. In brief, a diagnosis was made when the patients had at least five of the six major symptoms (fever, conjunctival congestion, oral mucosa alteration, cervical lymphadenopathy, swelling of extremities, and polymorphous rash), or when the patients had four major symptoms with the development of CAL. The first day of illness was defined as the day when at least one of the major symptoms appeared. Development of CAL was defined by quantifying the internal coronary artery dimension as per the Japanese Ministry of Health Criteria (a maximum absolute internal diameter > 3 mm in children < 5 years of age, or > 4 mm in children 5 years and older, or segment 1.5 times greater than an adjacent segment, or the presence of luminal irregularity) and whenever body surface area-adjusted *Z* score of any coronary artery was ≥ + 2.5 (including left main, left anterior descending, left circumflex arteries, and right coronary arteries) [2].The facilities included all of the 11 pediatric inpatient facilities in Yamanashi Prefecture and 1 facility in Nagano Prefecture in Japan. The registration database was constructed with anonymized clinical records of all the diagnosed KD cases in each hospital that were collected at the end of every year. During the COVID-19 pandemic (from March 2020 to December 2020), 44 cases were diagnosed with Kawasaki disease. In this 10-month period, COVID-19 was uncommon in Yamanashi Prefecture (only 22 children were diagnosed with COVID-19). None of these 44 cases were considered to be multisystem inflammatory syndrome in children (MIS-C) since all cases had no history of direct contact with people with COVID-19 cases within 4 weeks prior to diagnosis, and 37 cases were directly confirmed to be negative for SARS-CoV2 at admission (PCR analysis, 35 cases; antigen test, 2 cases). The study was performed under the approval by the Research Ethics Committee of University of Yamanashi Hospital (Approval Number 1698).

### Treatment of Kawasaki disease

Initial laboratory tests listed in Supplemental Table 2 had been standardized, and all of the patients were treated identically treated identically with a first-line regimen of 2 g/kg/dose of IVIG in combination with 30 mg/kg of oral aspirin immediately after the diagnosis of KD was made based on the above criteria. Standardized treatment workflow was confirmed in the meeting by each facility every year. IVIG therapy was completed within 24 h after diagnosis of KD in all of the patients. No patients were treated with glucocorticoids. The response to the initial treatment was evaluated 48 h after initiation of IVIG administration and was considered as “IVIG resistance” when the body temperature was over 37.5 °C, and the serum level of C-reactive protein (CRP) was higher than half of the peak value as previously reported [12, 22]. Body temperature was measured in the axilla using an electronic thermometer. IVIG-resistant patients were treated with second-line therapy comprising an additional 2 g/kg/dose of IVIG or 5 mg/kg of intravenous infliximab [23]. In addition, when the patients were considered to be resistant to the second-line therapy, plasma exchange was carried out after the patient was transferred to University of Yamanashi Hospital [24].

### Machine learning

The predictors for IVIG resistance were chosen from routinely available data including 6 demographic variables, 22 laboratory data, and 2 echocardiographic parameters at diagnosis as listed in Table 1. For any missing laboratory data and echocardiography parameter values, the median value was complementary used in the machine learning. We used the random forest model [25], eXtreme Gradient Boosting (XGBoost) [26], and light gradient boosting machine (Light GBM) [27], which are tree-based nonparametric methods requiring no assumption about data distribution. We also performed logistic regression analysis and support vector machine analysis (SVM) [28]. We operated each machine learning in the training set (approximately 80% of the random sample) using scikit-learn in Python software (version 3.8.3), and the optimal parameters (number of trees and the maximum depth of the tree) were determined according to the best area under the receiver operating characteristic (ROC) curve (AUC) in the validation set (approximately 20% of the random sample) as in the previous studies [14, 15] by using *k*-fold crossvalidation (*k* = 10) (Supplemental Fig. 1) [29]. Considering an imbalanced dataset of the IVIG response, we used synthetic minority over-sampling technique (SMOTE), which is a technique of over-sampling the minority class [30, 31].

**Table 1:** Variables in the model

Variables	
Clinical features	Sex, age in months, height, weight, days of illness at initial therapy (start day), patients with the absence or presence of five or more major symptoms
Laboratory data	White blood cell count (WBC); percentage of neutrophils (Neut); hemoglobin level (Hb); platelet count (Plt); serum levels of C-reactive protein (CRP), total protein (TP), albumin, globulin (IgG), sodium (Na), potassium (K), chloride (Cl), alanine aminotransferase (ALT), aspartate aminotransferase (AST), lactic acid dehydrogenase (LDH), total bilirubin (T. bilirubin), blood urea nitrogen (BUN), creatinine (Cre), creatine kinase (CK), total cholesterol (T. cholesterol), high-density lipoprotein cholesterol (HDL chol), and triglyceride(TG); D-dimer value
Echocardiographic parameters	Maximum coronary artery diameter before initial treatment (CA diameter), maximum coronary artery *Z* score before initial treatment (CA *Z* score)

### Development of the scoring system

For development of the simple scoring system to predict IVIG resistance, we selected the features that influenced the prediction model in the Light GBM algorithm, in which the highest AUC was observed. We used the Shapley additive explanation (SHAP), which is a unified approach for explaining the outcome of machine learning model [32–34]. SHAP values evaluate the importance of the output, and a higher SHAP value indicates that a feature has a larger impact and is more important on the model [15, 35]. To determine the cutoff level of each variable, we used the SHAP dependence plot, which evaluates significance of each feature in the output of the Light GBM model [19]. Based on the SHAP value, we constructed a new predictive scoring model (Yamanashi score). To validate the accuracy of the new score system, we applied the score system in the above Yamanashi study cohort and compared it with three previously established score systems.

### Statistical analysis

Statistical analyses were performed using EZR software (version 1.41) [36] and Python software (version 3.8.3). Spearman’s correlation coefficient was used to analyze the correlation of each score. Creation and comparison of the ROC curves were performed by using the EZR software.

## Results

### Prediction of IVIG resistance by machine learning

**Fig. 1:**
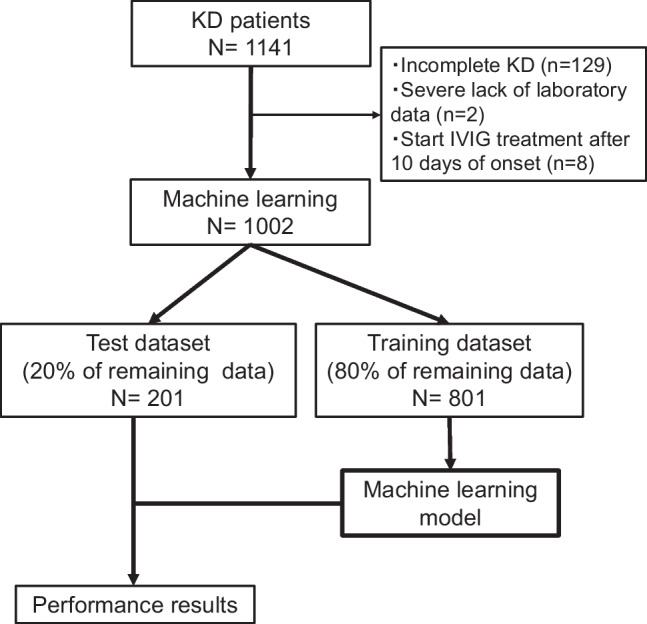
Flowchart of machine learning. Among 1141 consecutive Kawasaki disease cases enrolled in the cohort of the Yamanashi study, 139 cases were excluded for machine learning due to indicated reasons. The remaining 1002 cases were separated randomly into training dataset (80%, 801 cases) and test dataset (20%, 201 cases). Synthetic minority over-sampling technique (SMOTE) was applied for an imbalanced dataset of intravenous immunoglobulin (IVIG) resistance, and Shapley additive explanation (SHAP) was applied to identify the variables that influenced the prediction model

**Fig. 2:**
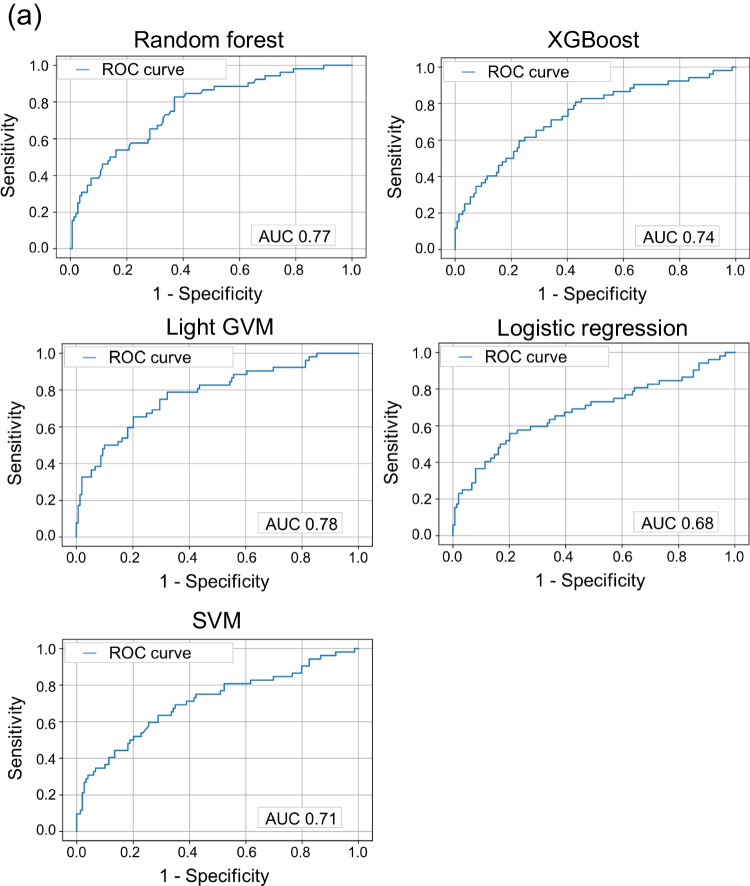

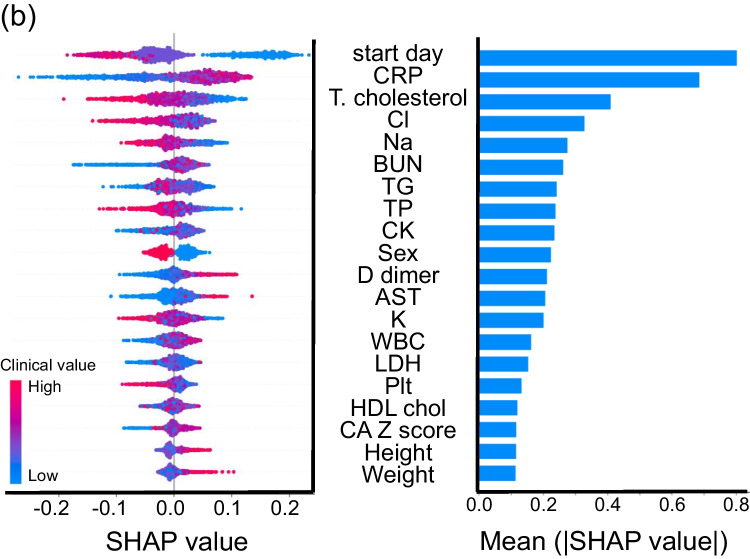
Prediction ability of machine learning for IVIG resistance. **a** ROC curves for IVIG resistance in five models. The horizontal axis indicates false positive rate (1 - specificity), and the vertical axis indicates true positive rate (sensitivity). AUC is indicated at the bottom. **b** The 20 most important features for predicting IVIG resistance in the Light GBM model. Left: SHAP summary plot of the top 20 features. The higher the SHAP value of a feature, the higher the probability of IVIG resistance. Each dot represents the feature attribution value of each patient. A red dot represents higher feature value, and a blue dot represents lower feature value. Right: Importance matrix plot of the top 20 features. SHAP value of each feature is indicated in descending order

From June 2010 to December 2020, 1141 consecutive KD cases were enrolled in the Yamanashi study cohort. In the present study, 139 cases were excluded for further analyses due to a diagnosis of incomplete KD (*n* = 129), severe lack of laboratory data (*n* = 2), or delayed IVIG treatment after 10 days of onset (*n* = 8) (Fig. 1). One hundred and ninety-three cases (19%) were diagnosed before day 5. In the remaining 1002 cases, 227 cases (22.7%) were resistant to the first course of IVIG treatment (demographics were indicated in Supplemental Tables 3 and 4). In the demographics of 12 facilities (Supplemental Table 5), variations in day of illness at initial therapy (median, 5.2 days; range, 4.9–5.8) and IVIG resistance (22.5%, 14–34%) were largely acceptable. We operated each machine learning using 6 demographic variables, 22 laboratory data, and 2 echocardiographic parameters at diagnosis listed in Table 1. The data of 1002 cases were divided at random into 801 cases of the training dataset (approximately 80%) and 201 cases of the test dataset (approximately 20%). Considering a relatively low frequency of IVIG resistance as an imbalanced dataset of machine learning, we applied SMOTE [30, 31]. Prediction values and ROC curves for IVIG resistance in each model are summarized in Table 2 and Fig. 2a. The highest AUC was observed in the Light GBM model (0.78) (Fig. 2a). In the Light GBM model, global accuracy, sensitivity, specificity, positive prediction value, negative prediction value, positive likelihood ratio, and negative likelihood ratio scores were 0.78 (95% confidence interval (CI), 0.72–0.84), 0.50 (0.36–0.64), 0.88 (0.82–0.93), 0.59 (0.43–0.74), 0.83 (0.77–0.89), 4.14 (2.48–6.90), and 0.57 (0.43–0.75), respectively. These observations demonstrated that machine learning models achieved good discriminating abilities to predict IVIG sensitivity in KD patients although the ability to predict IVIG resistance was relatively limited in this cohort.

**Table 2:** Prediction values in each machine learning model

	Random forest	XGBoost	Light GBM	Logistic regression	SVM
Global accuracy	0.77 (0.71–0.83)	0.76 (0.69–0.81)	0.78 (0.72–0.84)	0.72 (0.65–0.78)	0.70 (0.63–0.76)
Sensitivity	0.42 (0.29–0.57)	0.50 (0.36–0.64)	0.50 (0.36–0.64)	0.58 (0.43–0.71)	0.60 (0.45–0.73)
Specificity	0.89 (0.83–0.94)	0.85 (0.78–0.90)	0.88 (0.82–0.93)	0.77 (0.69–0.83)	0.73 (0.65–0.80)
Positive predictive value	0.58 (0.41–0.74)	0.53 (0.38–0.68)	0.59 (0.43–0.74)	0.46 (0.34–0.59)	0.44 (0.32–0.56)
Negative predictive value	0.82 (0.75–0.87)	0.83 (0.76–0.89)	0.83 (0.77–0.89)	0.84 (0.77–0.90)	0.84 (0.76–0.90)
Positive likelihood ratio	3.94 (2.25–6.90)	3.23 (2.04–5.15)	4.14 (2.48–6.90)	2.46 (1.69–3.56)	2.22 (1.57–3.14)
Negative likelihood ratio	0.65 (0.51–0.82)	0.59 (0.45–0.78)	0.57 (0.43–0.75)	0.55 (0.40–0.77)	0.55 (0.39–0.78)

95% confidence interval is indicated in parenthesis

### Development of scoring system to predict IVIG resistance

**Fig. 3:**
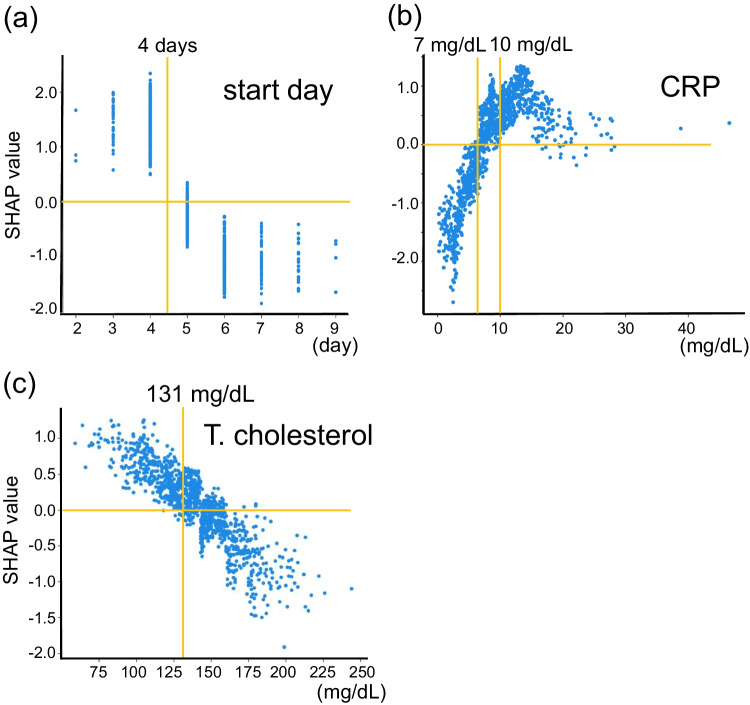
SHAP dependence plot of top three features. The SHAP dependence plots of **a** days of illness at initial therapy (start day), serum levels of **b** C-reactive protein (CRP), and **c** total cholesterol (T. cholesterol). The horizontal axis indicates the actual value of the feature, and the vertical axis indicates the SHAP value of the feature. The horizontal line indicates the zero level of the SHAP value, and the vertical line indicates the cutoff level for each variable. Cutoff value is indicated on the top in each panel

We next evaluated the top 20 features among 30 items tested in the Light GBM model using SHAP (Fig. 2b). In SHAP summary plot, the higher the SHAP value of a feature, the higher the probability of IVIG resistance. In each SHAP value of a feature, each dot represents the feature attribution value of each patient, and red and blue dots represent higher and lower feature value, respectively. The highest SHAP value feature was days of illness at initial therapy (start day) (SHAP value [average of absolute value], 0.80). Additionally, serum levels of CRP (0.68) and total cholesterol (0.41) were the other top two features. Base on the SHAP dependence plot of these three features (Fig. 3), we tried to create a new score system to predict IVIG resistance. The cutoff levels for each variable were determined based on the intersecting line of “zero” SHAP value (Fig. 3) as follows: start day ≤ day 4, CRP ≥ 10 and 7 mg/dL, and total cholesterol ≤ 131 mg/dL. Finally, we constructed a simple scoring model (Yamanashi score) using the three variables as follows: two points were scored for start day ≤ day 4 and CRP ≥ 10 mg/dL, while one point was given for CRP ≥ 7 mg/dL (and < 10 mg/dL) and total cholesterol ≤ 131 mg/dL. The maximum total score was five points.

### Validation of scoring systems to predict IVIG resistance

**Fig. 4:**
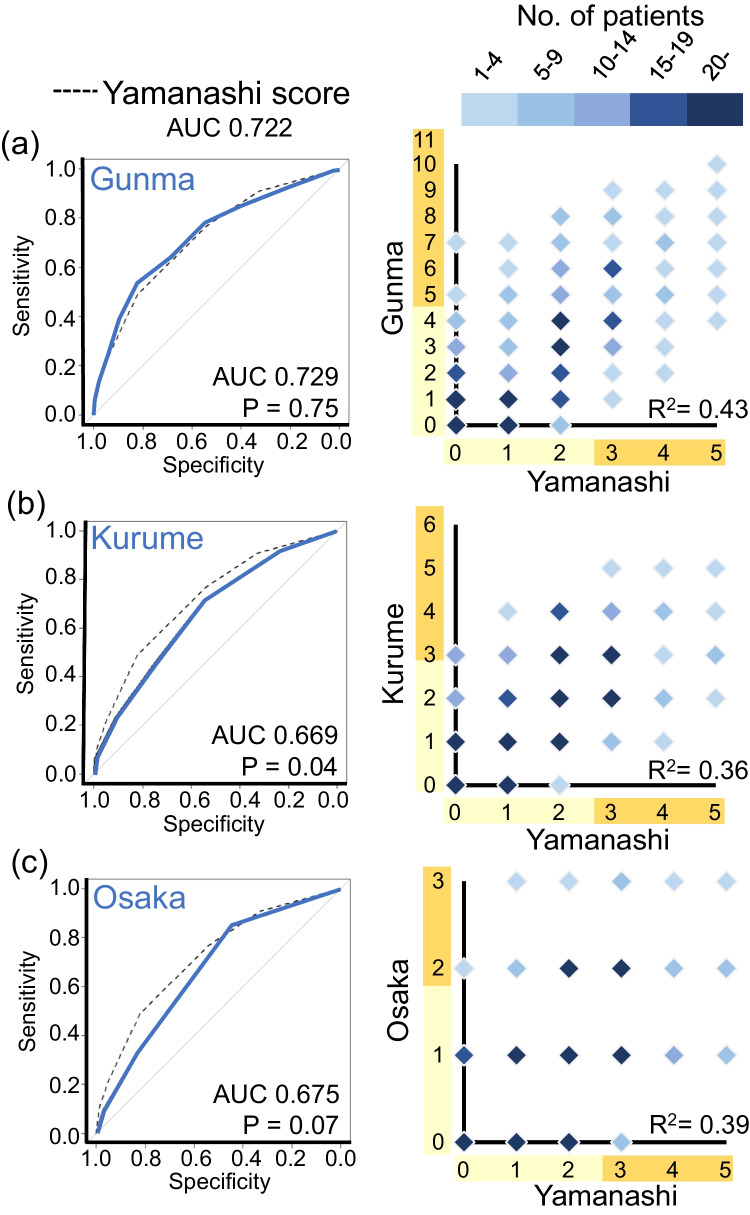
Comparison of Yamanashi score with three previously established scoring systems. Comparison of ROC curves (left panels) and correlations (right panels) between Yamanashi and Gunma (**a**), Kurume (**b**), and Osaka (**c**) scores in the 457 cases of the Yamanashi cohort study. Color scale indicates the number of cases

We validated the accuracy of the Yamanashi score in the prediction of IVIG resistance by comparing it with three representative scoring systems in the cohort of Yamanashi study. Among the 1002 KD cases, 545 cases were excluded for the validation due to lack of even one of variables for the 4 scoring systems, and thus the remaining 457 cases were available for further analyses. Among the 457 cases, 108 cases (23.6%) were resistant to initial IVIG treatment. Of note, when three points for the total score was applied as a cutoff (Supplemental Fig. 2), the accuracy (AUC, 0.72 (95%CI, 0.67–0.77); sensitivity, 0.49 (0.39–0.59); specificity, 0.82 (0.78–0.86)) of the Yamanashi score was almost identical to that (AUC, 0.78; sensitivity, 0.50; specificity, 0.88) of the Light GBM model using 30 clinical variables. Next, we compared the prediction accuracy of the Yamanashi score with three previous scoring systems (Fig. 4, Supplemental Table 6). Among three variables of the Yamanashi score, total cholesterol was not included in all of three previous scores, while start day and CRP were included in three and two previous scores, respectively (Supplemental Fig. 3). Interestingly, although only three variables were included in the Yamanashi score, ROC curve and AUC of the Yamanashi score were almost identical to those of the Gunma score (AUC, 0.73 (95% CI, 0.67–0.79)) (Fig. 4a), in which seven variables were included [11]. When the Gunma score was applied with a cutoff of five points for total score, sensitivity and specificity were 0.53 (95% CI, 0.43–0.63) and 0.83 (0.79–0.87), respectively. In the 457 cases of the Yamanashi cohort study, the Yamanashi score was significantly correlated with the Gunma score (*R*^2^ = 0.43). In Kurume (Fig. 4b) [12] and Osaka (Fig. 4c) [13] scores, although the correlation coefficients (*R*^2^) with the Yamanashi score were 0.36 and 0.39, respectively, AUC values (0.67 (95% CI, 0.61–0.73) and 0.68 (95% CI, 0.62–0.73)) were inferior (*p* = 0.04 and *p* = 0.07) to those of the Yamanashi score, respectively. These observations revealed that the simple scoring system using top three features in the machine learning model predicted IVIG resistance almost as accurately as the machine learning model itself as well as the widely used Gunma score, at least in the Yamanashi cohort study.

## Discussion

Recently established machine learning has been widely applied in the field of clinical medicine such as outcome prediction, diagnosis, and image interpretation [14–19]. In the present study, we applied the machine learning models to predict IVIG resistance of the initial KD treatment in the Yamanashi cohort study in which clinical data of the 1002 cases were available. Compared with the conventional model such as the widely used logistic regression model in which variables are selected by a knowledge-oriented approach, machine learning is an unbiased approach using a large number of variables. Taking advantage of machine learning, we applied all the initial laboratory data without any assumptions. Considering an imbalanced dataset of IVIG resistance, we applied SMOTE [30, 31] and confirmed a good discriminating ability to predict IVIG resistance. To apply the accurate prediction ability of machine learning model to clinical practice, we established a new scoring system (Yamanashi score) based on the findings in the SHAP plot [32–34] of the Light GBM model, which showed the best prediction accuracy among five models we tested. We selected the following three features with high SHAP values: days of illness at initial therapy, serum levels of CRP, and total cholesterol at diagnosis. Surprisingly, this simple scoring system using the only three features predicted IVIG resistance almost as accurately as the Light GBM model itself. Moreover, Yamanashi score was as reliable as three previously established major scoring systems [11–13]. Among the three features of Yamanashi score, two features were included in three scoring systems [11–13] as follows: serum CRP level was included in all three scoring systems (Gunma [11], Kurume [12], and Osaka [13]), and days of illness at initial therapy was included in two scoring systems (Gunma [11] and Kurume [12]). In contrast, serum total cholesterol level was not included in the three previously established scoring systems. These observations suggest that serum total cholesterol level may make a significant and unique contribution for an accurate prediction of the Yamanashi score.

In the SHAP dependence plot of the present study, lower serum total cholesterol level (cutoff value, 131 mg/dL) was associated with higher risk of IVIG resistance. Our finding seems to be consistent with a previous finding showing that levels of serum total cholesterol decreased in the acute phase of KD patients due to abnormal lipid metabolism [37]. In particular, recent report by Shao et al. [38] revealed that serum total cholesterol level before the initial IVIG treatment was significantly lower in the cases of IVIG resistance in a single-center prospective cohort study. Although the underlying mechanism for association between dyslipidemia and the severity of systemic inflammation in KD remains unclear, a recent study by Zhang et al. [39] revealed that dyslipidemia during acute phase of KD was associated with aberrant levels of adipokines including adiponectin, omentin-1, and chemerin. In the above study by Shao et al. [38], alterations in the other lipid proteins were also associated with IVIG resistance: a higher level of triglyceride and lower levels of high-density lipoprotein cholesterol, low-density lipoprotein cholesterol, and apolipoprotein A. Thus, although the lipid profile was not fully evaluated in the present study, dyslipidemia due to systemic inflammation in the acute phase of KD patients may be a rational explanation for the usefulness of serum total cholesterol level as one of predictors for IVIG resistance in the Yamanashi score.

Three previous studies applied machine learning to predict the IVIG resistance in the patients with Kawasaki disease [14–16]. However, no scoring systems were proposed in these previous reports. Moreover, in two previous reports [14, 16], a list of the top features was unavailable. In the previous report by Wang et al. [15], the top three features were reported to be platelet count, serum calcium level, and the ratio of serum albumin level to globulin level. Of note, in their SHAP values that were partially consistent with those in our study, days of fever, serum cholesterol level, and serum CRP level were listed as the fourth, eighth, and nineteenth features in the 20 most important features among 82 variables, respectively.

This study has several limitations. First, since the majority of the subjects in the present study were of Japanese ethnicities, further validation is required before the present scoring system can be applied to other ethnicities and different populations. Second, although the patients were treated with a standardized protocol, the study was based on retrospective data collection from a number of hospitals. Third, several known predictive factors such as neutrophil-to-lymphocyte and platelet-lymphocyte ratios [40] were not evaluated due to the lack of data collection. Recently, utilities of coagulation profile [41], hepcidin [42], and genetic variants of the interleukin gene [43] have been also reported. Thus, machine learning using these factors as additional variables might improve the accuracy. Feature engineering of clinical variables is another possibility to further improve the accuracy [44]. Forth, insufficient reduction in the serum CRP level was additionally included in the definition of IVIG resistance in the present study as previously reported by others [12, 22], while only persistent fever was evaluated in many studies [45, 46]. Fifth, external validation for the practical use of our scoring system might be difficult since serum cholesterol level is not routinely evaluated in the other cohorts. In this context, however, when we applied this system to the recent 73 cases (IVIG resistance, 14 cases) who were diagnosed in the same 12 facilities from January 2021 to May 2022 and confirmed them to be negative for SARS-CoV2 at diagnosis by PCR or antigen test, prediction values in this system were similar to those in the previous three scoring systems (Supplemental Table 7) [11–13].

In conclusion, we implemented the machine learning algorithm to predict IVIG resistance in KD patients and confirmed its potential. Moreover, using only three features of the machine learning model, we designed a simple scoring system to predict IVIG resistance. Of note, in spite of its simplicity, the scoring system predicted IVIG resistance almost as accurately as the machine learning approach as well as three previously established major scoring systems.

## Supplementary Information

Below is the link to the electronic supplementary material.

Supplementary file1 Supplemental Table 1. List of facilities (PDF 11.2 KB)

Supplementary file2 Supplemental Table 2. Standardized pre-treatment clinical examinations (PDF 63.3 KB)

Supplementary file3 Supplemental Table 3. Comparison of the baseline demographics and clinical features of patients who are IVIG responsive and resistant in the training data (PDF 65.1 KB)

Supplementary file4 Supplemental Table 4. Comparison of the baseline demographics and clinical features of patients who are IVIG responsive and resistant in the test data (PDF 18.6 KB)

Supplementary file5 Supplemental Table 5. Demographics in each facility (PDF 88.3 KB)

Supplementary file6 Supplemental Table 6. Prediction values in each score (PDF 177 KB)

Supplementary file7 Supplemental Table 7. Accuracy of each score for 73 KD patients with ruled out MIS-C by COVID-19 from January 2021 to May 2022 (PDF 126 KB)

Supplementary file8 Supplemental Figure 1. Flowchart of k-fold cross validation. The data of 1002 cases were divided at random into training dataset (approximately 80%) and test dataset (approximately 20%). The generalization performance of training dataset was evaluated by stratified k-fold cross validation (k=10). (PDF 9.01 KB)

Supplementary file9 Supplemental Figure 2. IVIG resistance rate in each new score (Yamanashi score). When three points for the total score was applied as a cutoff, AUC of the Yamanashi score was 0.72 (95%CI: 0.67 – 0.77), sensitivity was 0.49 (0.39 – 0.59) and specificity was 0.82 (0.78 – 0.86). (PDF 14.7 KB)

Supplementary file10 Supplemental Figure 3. Variables for each score. Yamanashi score consisted of three variables, while the Gunma, Kurume, and Osaka scores consisted of seven, five, and three variables, respectively. Total cholesterol level was not included in Gunma, Kurume, or Osaka score. (PDF 64.0 KB)

## Data Availability

The datasets generated during and analyzed during the current study are not publicly available due to the risk of revealing the identity of the subjects but are available from the corresponding author on reasonable request.

## Abbreviations

AUC: Area under the ROC curve
CAL: Coronary artery lesions
CRP: C-reactive protein
IVIG: Intravenous immunoglobulin
KD: Kawasaki disease
Light GBM: Light gradient boosting machine
ROC: Receiver operating characteristic
SHAP: Shapley additive explanation
SMOTE: Synthetic minority over-sampling technique
