# Correction to: A simple scoring model based on machine learning predicts intravenous immunoglobulin resistance in Kawasaki disease

**DOI:** 10.1007/s10067-023-06555-2

**Published:** 2023-03-15

**Authors:** Yuto Sunaga, Atsushi Watanabe, Nobuyuki Katsumata, Takako Toda, Masashi Yoshizawa, Yosuke Kono, Yohei Hasebe, Keiichi Koizumi, Minako Hoshiai, Eiryo Kawakami, Takeshi Inukai

**Affiliations:** 1 Faculty of Medicine, Department of Pediatrics, University of Yamanashi, 1110 Shimokato, Chuo, Yamanashi 409-3898 Japan; 2 Department of Neonatology, Yamanashi Prefectural Central Hospital, Kofu, Yamanashi Japan; 3 Department of Pediatric Cardiology, National Cerebral and Cardiovascular Center, Osaka, Japan; 4 Department of Pediatrics, Fujiyoshida Municipal Hospital, Fujiyoshida, Yamanashi Japan; 5 Department of Pediatrics, Yamanashi Prefectural Central Hospital, Kofu, Yamanashi Japan; 6 Artificial Intelligence Medicine, Graduate School of Medicine, Chiba University, Chiba, Japan; 7 Institute for Advanced Academic Research (IAAR), Chiba University, Chiba, Japan; 8 Advanced Data Science Project, RIKEN Information R&D and Strategy Headquarters, Kanagawa, Japan

**Correction to: Clinical Rheumatology**

10.1007/s10067-023-06502-1

The Supplementary materials in the original published version of the above article were incorrectly uploaded to their corresponding legends. The material has been reuploaded under the correct legends.

The original article has been corrected.

