# Supplementary material for: A simple scoring model based on machine learning predicts intravenous immunoglobulin resistance in Kawasaki disease
Source: Clin Rheumatol. 2023 Jan 11;42(5):1351–61. doi: 10.1007/s10067-023-06502-1 (PMC9832252; doi:10.1007/s10067-023-06502-1)
Supplement: Supplementary file 1 — Supplementary file1 Supplemental Table 1. List of facilities (PDF 11.2 KB) [file 10067_2023_6502_MOESM1_ESM.pdf]

Supplemental Table 1. List of facilities

**Facilities**	No. of cases
Yamanashi Prefectural Central Hospital	238
Kofu Municipal Hospital	191
Yamanashi Kosei Hospital	172
National Hospital Organization Kofu National Hospital	113
Suwa Central Hospital	86
Yamanashi Red Cross Hospital	86
Fujiyoshida Municipal Hospital	65
Nirasaki City Hospital	61
Kofu-Kyoritsu Hospital	47
Tsuru Municipal General Hospital	42
Kyonan Medical Center Fujikawa Hospital	32
University of Yamanashi	8
