# Supplementary material for: A simple scoring model based on machine learning predicts intravenous immunoglobulin resistance in Kawasaki disease
Source: Clin Rheumatol. 2023 Jan 11;42(5):1351–61. doi: 10.1007/s10067-023-06502-1 (PMC9832252; doi:10.1007/s10067-023-06502-1)
Supplement: Supplementary file 2 — Supplementary file2 Supplemental Table 2. Standardized pre-treatment clinical examinations (PDF 63.3 KB) [file 10067_2023_6502_MOESM2_ESM.pdf]

Supplemental Table 2. Standardized pre-treatment clinical examinations

☐ Blood test data	White blood cell count (WBC); percentage of neutrophils (Neut); hemoglobin level (Hb); platelet count (Plt); serum levels of C-reactive protein (CRP), total protein (TP), albumin, globulin (IgG), sodium(Na), potassium(K), chloride (Cl),alanine aminotransferase (ALT), aspartate aminotransferase (AST), lactic acid dehydrogenase (LDH), total bilirubin (T. bilirubin),blood urea nitrogen (BUN), creatinine (Cre), creatine kinase (CK), total cholesterol (T. cholesterol), high-density lipoprotein cholesterol (HDL chol), and triglyceride(TG); D-dimer value
☐ echocardiography	
☐ Twelve-lead electrocardiography	
☐ Chest X-ray examination	

Blood tests and echocardiography are performed to evaluate response to treatment. The above examinations should be reconfirmed as much as possible prior to discharge.
