# Supplementary material for: A simple scoring model based on machine learning predicts intravenous immunoglobulin resistance in Kawasaki disease
Source: Clin Rheumatol. 2023 Jan 11;42(5):1351–61. doi: 10.1007/s10067-023-06502-1 (PMC9832252; doi:10.1007/s10067-023-06502-1)
Supplement: Supplementary file 3 — Supplementary file3 Supplemental Table 3. Comparison of the baseline demographics and clinical features of patients who are IVIG responsive and resistant in the training data (PDF 65.1 KB) [file 10067_2023_6502_MOESM3_ESM.pdf]

Supplemental Table 3. Comparison of the baseline demographics and clinical features of patients who are IVIG responsive and resistant in the training data in the test data

	IVIG response (n=626)	IVIG resistance (n=175)	P value
Age(month); mean(SD)	32 ± 25	34 ± 24	0.28
range (month)	0 – 154	1 - 131	
Male; %	56	55.9	0.91
Height(cm); mean(SD)	87 ± 12	88 ± 13	0.39
Weight(kg); mean(SD)	12 ± 4.0	13 ± 3.8	0.44
Percentage of patients with five or more major symptoms; %	99	99	0.98
1st line start day; mean(SD)	5.3 ± 1.0	4.9 ± 1.0	< 0.05
Percentage of patients diagnosed before day 5; %	14.3	33.7	< 0.05
