# Supplementary material for: A simple scoring model based on machine learning predicts intravenous immunoglobulin resistance in Kawasaki disease
Source: Clin Rheumatol. 2023 Jan 11;42(5):1351–61. doi: 10.1007/s10067-023-06502-1 (PMC9832252; doi:10.1007/s10067-023-06502-1)
Supplement: Supplementary file 4 — Supplementary file4 Supplemental Table 4. Comparison of the baseline demographics and clinical features of patients who are IVIG responsive and resistant in the test data (PDF 18.6 KB) [file 10067_2023_6502_MOESM4_ESM.pdf]

Supplemental Table 4. Comparison of the baseline demographics and clinical features of patients who are IVIG responsive and resistant in the test data

	IVIG response (n=149)	IVIG resistance (n=52)	P value
Age(month); mean(SD)	32±23	31±23	0.87
range (month)	4 - 151	3 - 135	
Male; %	49	54	0.56
Height(cm); mean(SD)	88±12	90±13	0.41
Weight(kg); mean(SD)	13±3.6	12±3.6	0.88
Percentage of patients with five or more major symptoms; %	100	98	0.32
1st line start day; mean(SD)	5.3±1.0	4.7±1.0	< 0.05
Percentage of patients diagnosed before day 5; %	14.0	44.2	< 0.05
