# Supplementary material for: A simple scoring model based on machine learning predicts intravenous immunoglobulin resistance in Kawasaki disease
Source: Clin Rheumatol. 2023 Jan 11;42(5):1351–61. doi: 10.1007/s10067-023-06502-1 (PMC9832252; doi:10.1007/s10067-023-06502-1)
Supplement: Supplementary file 5 — Supplementary file5 Supplemental Table 5. Demographics in each facility (PDF 88.3 KB) [file 10067_2023_6502_MOESM5_ESM.pdf]

Supplemental Table 5. Demographics in each facility

Facilities	No. of cases	Age (month), mean(SD)	Male,%	1st line start day, mean(SD)	IVIG resistance, %
Yamanashi Prefectural Central Hospital	216	30 (25)	52	5.4 (0.9)	16
Kofu Municipal Hospital	171	32 (27)	53	5.2 (1.2)	20
Yamanashi Kosei Hospital	155	35 (28)	57	4.9 (1.0)	34
National Hospital Organization Kofu National Hospital	100	30 (21)	57	5.0 (1.0)	23
Suwa Central Hospital	82	38 (27)	62	5.3 (0.9)	17
Yamanashi Red Cross Hospital	69	28 (20)	54	5.0 (1.0)	22
Nirasaki City Hospital	52	38 (22)	56	5.2 (0.9)	29
Fujiyoshida Municipal Hospital	49	35 (27)	51	5.2 (0.7)	33
Kofu-Kyoritsu Hospital	42	25 (20)	50	5.5 (0.9)	17
Tsuru Municipal General Hospital	30	31 (21)	50	5.0 (0.7)	33
Kyonan Medical Center Fujikawa Hospital	28	33 (21)	64	5.1 (1.2)	14
University of Yamanashi	8	28 (34)	88	5.8 (1.4)	25
