# Supplementary material for: A simple scoring model based on machine learning predicts intravenous immunoglobulin resistance in Kawasaki disease
Source: Clin Rheumatol. 2023 Jan 11;42(5):1351–61. doi: 10.1007/s10067-023-06502-1 (PMC9832252; doi:10.1007/s10067-023-06502-1)
Supplement: Supplementary file 6 — Supplementary file6 Supplemental Table 6. Prediction values in each score (PDF 177 KB) [file 10067_2023_6502_MOESM6_ESM.pdf]

Supplemental Table 6. Prediction values in each score

	Scoring system			
	Yamanashi	Gunma	Kurume	Osaka
Global accuracy	0.74<br>(0.70 - 0.78)	0.76<br>(0.72 - 0.80)	0.68<br>(0.64 - 0.73)	0.72<br>(0.67 - 0.76)
Sensitivity	0.49<br>(0.39 - 0.59)	0.53<br>(0.43, 0.63)	0.45<br>(0.36 - 0.55)	0.32<br>(0.24 - 0.42)
Specificity	0.82<br>(0.78 - 0.86)	0.83<br>(0.79 - 0.87)	0.75<br>(0.71 - 0.80)	0.84<br>(0.80 - 0.88)
Positive predictive value	0.46<br>(0.37 - 0.56)	0.49<br>(0.40 - 0.59)	0.36<br>(0.28 - 0.45)	0.39<br>(0.28 - 0.49)
Negative predictive value	0.84<br>(0.80 - 0.88)	0.85<br>(0.81 - 0.89)	0.82<br>(0.77 - 0.86)	0.80<br>(0.76 - 0.84)
Positive likelihood ratio	2.76<br>(2.05 – 3.72)	3.12<br>(2.33 - 4.19)	1.84<br>(1.40 - 2.43)	2.02<br>(1.41 - 2.90)
Negative likelihood ratio	0.62<br>(0.51 - 0.75)	0.57<br>(0.46 - 0.70)	0.73<br>(0.60 - 0.87)	0.81<br>(0.70 - 0.93)
AUC	0.72<br>(0.67 - 0.77)	0.73<br>(0.67 - 0.79)	0.67<br>(0.61 - 0.73)	0.68<br>(0.62 - 0.73)

95% confidence interval is indicated in parenthesis.
