# Supplementary material for: A simple scoring model based on machine learning predicts intravenous immunoglobulin resistance in Kawasaki disease
Source: Clin Rheumatol. 2023 Jan 11;42(5):1351–61. doi: 10.1007/s10067-023-06502-1 (PMC9832252; doi:10.1007/s10067-023-06502-1)
Supplement: Supplementary file 7 — Supplementary file7 Supplemental Table 7. Accuracy of each score for 73 KD patients with ruled out MIS-C by COVID-19 from January 2021 to May 2022 (PDF 126 KB) [file 10067_2023_6502_MOESM7_ESM.pdf]

Supplemental Table 7. Accuracy of each score for 73 KD patients with ruled out MIS-C by COVID-19 from January 2021 to May 2022

	Scoring system			
	Yamanashi	Gunma	Kurume	Osaka
Global accuracy	0.66 (0.54 - 0.77)	0.73 (0.61 - 0.82)	0.67 (0.55 - 0.78)	0.75 (0.64 - 0.85)
Sensitivity	0.43 (0.18 - 0.71)	0.36 (0.13 - 0.65)	0.29 (0.08 - 0.58)	0.36 (0.13 - 0.65)
Specificity	0.71 (0.58 - 0.82)	0.81 (0.69 - 0.90)	0.76 (0.63 - 0.86)	0.85 (0.73 - 0.93)
Positive predictive value	0.26 (0.10 - 0.48)	0.31 (0.11 - 0.59)	0.22 (0.06 - 0.48)	0.36 (0.13 - 0.65)
Negative predictive value	0.84 (0.71 - 0.93)	0.84 (0.72 - 0.93)	0.82 (0.69 - 0.91)	0.85 (0.73 - 0.93)
Positive likelihood ratio	1.48 (0.72 – 3.07)	1.92 (0.79 - 4.63)	1.20 (0.47 – 3.10)	2.34 (0.93 – 5.90)
Negative likelihood ratio	0.80 (0.50 – 1.30)	0.79 (0.53 – 1.19)	0.94 (0.65 – 1.34)	0.76 (0.51 – 1.14)
