# Supplementary figures and images for: A simple scoring model based on machine learning predicts intravenous immunoglobulin resistance in Kawasaki disease
Source: Clin Rheumatol. 2023 Jan 11;42(5):1351–61. doi: 10.1007/s10067-023-06502-1 (PMC9832252; doi:10.1007/s10067-023-06502-1)

Machine learning  
N= 1002

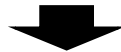

Test (20%)      Training (80%)

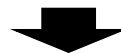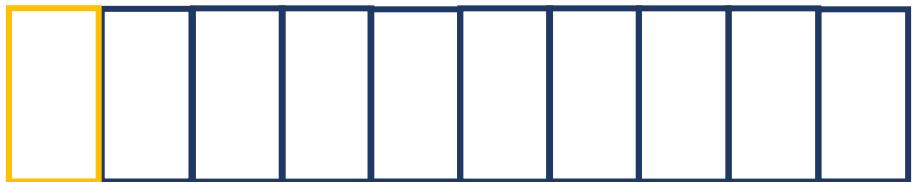

Validation fold

Training fold

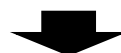

Average 0.86 (range: 0.77-0.97)

Supplement: Supplementary file 8 — Supplementary file8 Supplemental Figure 1. Flowchart of k-fold cross validation. The data of 1002 cases were divided at random into training dataset (approximately 80%) and test dataset (approximately 20%). The generalization performance of training dataset was evaluated by stratified k-fold cross validation (k=10). (PDF 9.01 KB) [file 10067_2023_6502_MOESM8_ESM.pdf]
