# Supplementary material for: A simple scoring model based on machine learning predicts intravenous immunoglobulin resistance in Kawasaki disease
Source: Clin Rheumatol. 2023 Jan 11;42(5):1351–61. doi: 10.1007/s10067-023-06502-1 (PMC9832252; doi:10.1007/s10067-023-06502-1)
Supplement: Supplementary file 9 — Supplementary file9 Supplemental Figure 2. IVIG resistance rate in each new score (Yamanashi score). When three points for the total score was applied as a cutoff, AUC of the Yamanashi score was 0.72 (95%CI: 0.67 – 0.77), sensitivity was 0.49 (0.39 – 0.59) and specificity was 0.82 (0.78 – 0.86). (PDF 14.7 KB) [file 10067_2023_6502_MOESM9_ESM.pdf]

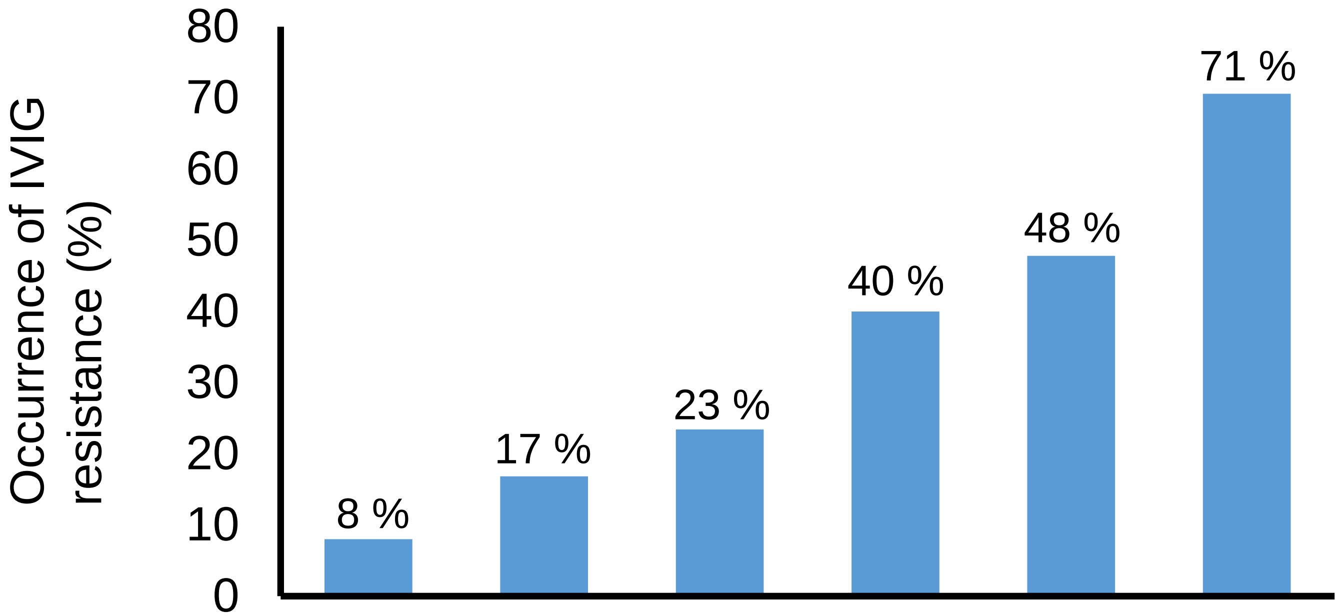

Yamanashi score	0	1	2	3	4	5
Number (n)	125	89	128	75	23	17
IVIG resistant (n)	10	15	30	30	11	12
sensitivity	1.0	0.91 (0.84-0.96)	0.77 (0.68-0.84)	0.49 (0.39-0.59)	0.21 (0.14-0.30)	0.11 (0.06-0.19)
specificity	0.0	0.33 (0.28-0.38)	0.54 (0.49-0.60)	0.82 (0.78-0.86)	0.95 (0.92-0.97)	0.99 (0.97-1.00)

95% confidence interval is indicated in parenthesis.
