# Supplementary material for: A simple scoring model based on machine learning predicts intravenous immunoglobulin resistance in Kawasaki disease
Source: Clin Rheumatol. 2023 Jan 11;42(5):1351–61. doi: 10.1007/s10067-023-06502-1 (PMC9832252; doi:10.1007/s10067-023-06502-1)
Supplement: Supplementary file 10 — Supplementary file10 Supplemental Figure 3. Variables for each score. Yamanashi score consisted of three variables, while the Gunma, Kurume, and Osaka scores consisted of seven, five, and three variables, respectively. Total cholesterol level was not included in Gunma, Kurume, or Osaka score. (PDF 64.0 KB) [file 10067_2023_6502_MOESM10_ESM.pdf]

Yamanashi score
2 point : first line start day ≤ 4 days
2 point : CRP ≥ 10 mg/dL
1 point : 10> CRP ≥ 7 mg/dL
1 point : T. cholesterol ≤ 131 mg/dL
Cutoff score ; 3 points

Kurume score
2 point : ALT ≥ 80 IU/L,
1 point : first line start day ≤ 4 days
1 point : CRP ≥ 8 mg/dL
1 point : Plt ≤ 30×10^4 /mm^3
1 point : Age ≤ 6 month
Cutoff score ; 3 points

Gunma score
2 point : Na ≤ 133 mmol/L
2 point : AST ≥ 100 IU/L
2 point : first line start day ≤ 4 days
2 point : Neutrophil ≥ 80 %
1 point : CRP ≥ 10 mg/dL
1 point : Plt ≤ 30×10^4 /mm^3
1 point : Age ≤ 12 month
Cutoff score ; 5 points

Osaka score
1 point : T. bilirubin ≥ 0.9 mg/dL
1 point : AST ≥ 200 IU/L
1 point : CRP ≥ 7 mg/dL
Cutoff score ; 2 points
